# Improvement of the Targeting of Radiolabeled and Functionalized Liposomes with a Two-Step System Using a Bispecific Monoclonal Antibody (Anti-CEA × Anti-DTPA–In)

**DOI:** 10.3389/fmed.2015.00083

**Published:** 2015-11-25

**Authors:** Aurore Rauscher, Mathieu Frindel, Holisoa Rajerison, Sébastien Gouard, Catherine Maurel, Jacques Barbet, Alain Faivre-Chauvet, Marie Mougin-Degraef

**Affiliations:** ^1^Centre de Recherche en Cancérologie Nantes-Angers (CRCNA), 6299 CNRS, UMR 892 – INSERM, Université de Nantes, Nantes, France; ^2^Nuclear Medicine Department, Institut de Cancérologie de l’Ouest, Saint Herblain, France; ^3^Nuclear Medicine Department, University Hospital Nantes, Nantes, France; ^4^GIP Arronax, Saint Herblain, France

**Keywords:** pegylated liposomes, radioimmunotherapy, bispecific antibody, pretargeting, solid tumors

## Abstract

This study proposes liposomes as a new tool for pretargeted radioimmunotherapy (RIT) in solid tumors. Tumor pretargeting is obtained by using a bispecific monoclonal antibody [BsmAb, anti-CEA × anti-DTPA-indium complex (DTPA–In)] and pegylated radioactive liposomes containing a lipid-hapten conjugate (DSPE–PEG–DTPA–In). In this work, the immunospecificity of tumor targeting is demonstrated both *in vitro* by fluorescence microscopy and *in vivo* by biodistribution studies.

**Methods:**

Carcinoembryonic antigen (CEA)-expressing cells (LS174T) were used either in cell culture or as xenografts in nude mice. Doubly fluorescent liposomes or doubly radiolabeled liposomes were, respectively, used for *in vitro* and *in vivo* studies. In each case, a tracer of the lipid bilayer [rhodamine or indium-111 (^111^In)] and a tracer of the aqueous phase [fluorescein or iodine-125 (^125^I)] were present. The targeting of liposomes was assessed with BsmAb for active targeting or without for passive targeting.

**Results:**

Data obtained with the lipid bilayer tracer showed a fluorescent signal on cell membranes two to three times higher for active than for passive targeting. This immunospecificity was confirmed *in vivo* with tumor uptake of 7.5 ± 2.4% ID/g (percentage of injected dose per gram of tissue) for active targeting versus 4.5 ± 0.45% ID/g for passive targeting (*p* = 0.03). Regarding the aqueous phase tracer, results are slightly more contrasted. *In vitro*, the fluorescent tracer seems to be released in the extracellular matrix, which can be correlated with the *in vivo* data. Indeed, the tumor uptake of ^125^I is lower than that of ^111^In: 5.1 ± 2.5% ID/g for active targeting and 2.7 ± 0.6% ID/g for passive targeting, but resulted in more favorable tumor/organs ratios.

**Conclusion:**

This work demonstrated the tumor targeting immunospecificity of DSPE–PEG–DTPA–In liposomes by two different methods. This original and new approach suggests the potential of immunospecific targeting liposomes for the RIT of solid tumors.

## Introduction

The main purpose of radioimmunotherapy (RIT) is to kill cancer cells by immunospecific targeting radionuclides to specific antigens expressed at their surface. It has been demonstrated to be effective for the treatment of hematologic malignancies using directly radiolabeled antibodies targeting differentiation antigens, particularly in the treatment of malignant B cell lymphomas (1, 2). But despite promising results, RIT is not as successful against solid malignancies, which are usually more radioresistant and less accessible to radiolabeled antibodies (3). Higher absorbed doses are necessary but administered activity is limited by normal organ toxicities (4).

In order to optimize the RIT of solid tumors, multi-step techniques, referred to as pretargeting, have been developed to improve target-to-normal tissues ratios and increase administered activities while limiting healthy organ exposure. One of the pretargeting approaches is the affinity enhancement system (AES), which is based on bispecific monoclonal antibodies (BsmAb) and radiolabeled bivalent haptens (5). This approach has been extensively tested in the clinic and has shown an increase of overall survival of patients with progressive metastatic Medullary Thyroid Carcinoma, which provided the first evidence of survival improvement by RIT in solid tumors (6, 7). Nevertheless, the radiolabeled small molecular bivalent haptens are limited for RIT by the amount of activity they can carry in practice (8). Up to now, the specific activity of developed haptens labeled with available radionuclides does not exceed 100–150 MBq/nmol and even less for radionuclides with a long half-life, which limits the activity delivered to tumor cells. In this context, liposomes, which up to now have been especially developed for drug targeting, can represent a new and original method of radiotherapy of cancers. The development of imaging applications with radioactive liposomes is widely described (9, 10), but their advantages for carrying therapeutic radionuclides for cancer therapy could be further exploited.

This study aims at using immunospecific radiolabeled liposomes for RIT of solid tumors because they offer the possibility to carry high radionuclide activities, by radiolabeling their membrane, the inner aqueous phase or both. The potentially high number of radioactive atoms carried by each liposome should increase the dose delivered to the tumor and the avidity effect expected from the multiplicity of the liposome – target cell connections should increase activity accretion in tumors, as with the AES that uses bivalent haptens to achieve cooperative binding to target cells. Finally, this liposome targeting approach will allow us to obtain an intermediate system between the direct targeting of radiolabeled mAb, which expose normal tissues, especially the red bone marrow, to excessive radiations and cause hematologic toxicities, and pretargeting which improves the tumor to background activity ratios but with a limitation in the activity that haptens can deliver to tumor cells.

**Figure 1:**
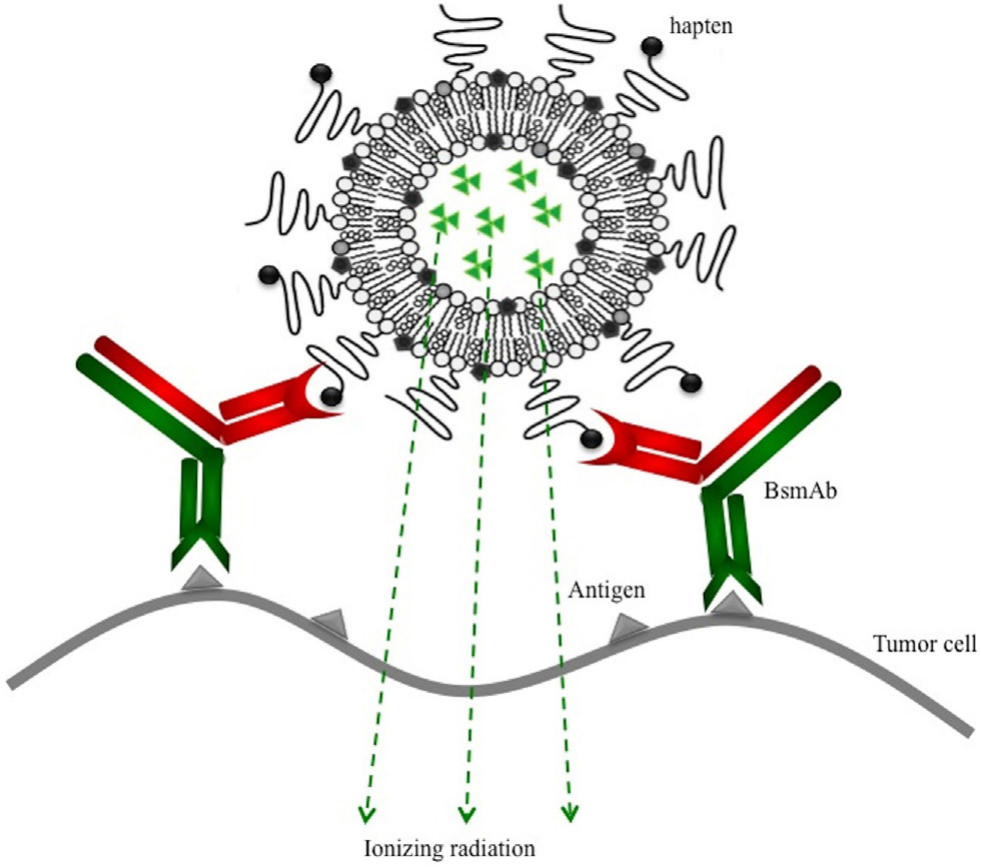
**Pretargeting of radiolabeled and functionalized liposomes using a bispecific monoclonal antibody (BsmAb) and a lipid-hapten conjugate**.

In this paper, we aim to target tumors, by a two-step approach, using functionalized liposomes and a BsmAb (hMN14 × 734) that recognizes on one arm the carcinoembryonic antigen (CEA) and on the other the DTPA–indium complex (DTPA–In). We used PEGylated liposomes that have been shown to be capable of very long circulation after intravenous injection with the hapten (DTPA–In) coupled at the end of the PEG chains (Figure 1). Surface plasmon resonance (SPR) was used previously to characterize specific interactions between antibodies and functionalized liposomes in order to select the best formulation in terms of hapten presentation and density of PEG chains (11). The chosen formulation optimizes the antibody-hapten recognition by orienting the haptens away from the PEG structure (apparent dissociation constant *K_*D*_* = 6.3 × 10^−9^M), while keeping a long half-life *in vivo* (*T*_1/2_ = 12.5 h). The functionalized liposomes present a large number of hapten molecules at their surface (around 400), favorable pharmacokinetic parameters, and the possibility to be radiolabeled at high specific activities. For that, an original method to label preformed liposomes was previously developed, that could be extended to radionuclides of interest for therapy (iodine-131 or astatine-211). This technique is based on the use of radioiodinated Bolton-Hunter reagent (BH) and liposomes containing high concentration of arginine in order to radiolabel the inner aqueous core with ^125^I (12). The liposome membrane was also radiolabeled with ^111^In, using a chelating lipid inserted in the bilayer (DSPE–PEG–DTPA). The DTPA–Indium complex formed is the hapten specifically recognized by the 734 arm of the hMN14 × 734 bispecific antibody.

Then, specific tumor targeting experiments were performed *in vitro*, by fluorescence microscopy, on CEA-expressing cells (LS734T), and *in vivo* in nude mice xenografted with human colorectal adenocarcinoma cells (LS734T) to demonstrate the immunospecifity of pretargeting using functionalized liposomes and a bispecific antibody. Doubly fluorescent liposomes or doubly radiolabeled liposomes were, respectively, used for *in vitro* and *in vivo* studies. The double radiolabeling, with a tracer of the lipid bilayer (rhodamine or ^111^In) and a tracer of the aqueous phase (fluorescein or ^125^I), was very useful to monitor the liposome integrity during the experiments and to know the behavior of the liposome contents.

## Materials and Methods

### Reagents

*N*-succinimidyl-3-(4-hydoxyphenyl)-propionate (BH) was purchased from Pierce Chemical Co. (Rockford, IL, USA). Arginine, chloramine T, fluorescein isothiocyanate (FITC), 1,2-distearoyl-glycero-3-phosphocholine (DSPC), and cholesterol (Chol) were from Sigma-Aldrich (Steinheim, Germany).

1,2-Distearoyl-sn-glycerol-3-phophoethanolamine-*N*-[Methoxy(Poly-ethylene glycol)-2000], M.W: 2805.54 (DSPE–PEG2000) and 1,2-dioleoyl-sn-glycero-3-phosphoethanolamine-*N*-(lissamine rhodamine B sulfonyl) M.W: 1301,7 (DPPE-rhodamine) were purchased from Avanti Polar Lipids (Alabaster, AL, USA). DSPE–PEG2000–DTPA was synthesized by Ecole Nationale Supérieure de Chimie de Rennes (France).

Vesicle extruder and filter supports were purchased from Avanti Polar Lipids, Inc. Polycarbonate membranes for vesicle extrusion (100 nm pore size, Nucleopore) were from Whatman. All phospholipids were dissolved in 9:1 chloroform/methanol mixture (HPLC grade, Carlo Erba, and Fisher Scientific).

The anti-CEA/anti-DTPA–In (hMN14 × 734) BsmAb was kindly provided by IBC Pharmaceuticals, Inc. (Morris Plains, NJ, USA).

Stable indium chloride (^115^In) was purchased from Sigma-Aldrich and radioactive indium-111 chloride (^111^In) was purchased from Covidien (Petten, The Netherlands). ^125^I-iodide sodium was purchased from Perkin-Elmer (Wellesley, MA, USA).

### Liposome Preparation and Characterization

DSPC/Chol/DSPE–PEG2000/DSPE–PEG2000–DTPA (64,5:30,5:3,5:1,5 molar ratio) or DSPC/Chol/DSPE–PEG2000/DSPE–PEG2000–DTPA/DPPE–Rhodamine (64:30,5:3,5:1,5:0,5 molar ratio) liposomes were prepared according to the lipid film hydration method described by Bangham (13).

Briefly, a total of 20 μmol of lipids (according to the molar ratios) was dissolved in chloroform/methanol (9:1 *v*/*v*) in a round bottom flask. A thin dry lipid film was obtained by solvent evaporation using a rotary evaporator (Rotavapor^®^, Buchi). Hydration of the lipid film was performed by addition of 1 ml aqueous phase. The flask was vortexed vigorously and maintained above the transition temperature of lipids during 2 h in a rotary evaporator without vacuum at 74°C (gel-crystal transition temperature of DSPE). The final concentration of the liposome suspension was 20 μmol of lipids/ml.

The aqueous phase was composed of 80 mM HEPES buffer pH 8 containing 80 mM arginine for radioactive liposomes and arginine 80 mM/HEPES 80 mM/FITC 20 mM for the fluorescent liposomes.

To obtain small and homogeneous vesicles, the liposome suspension was extruded through Nucleopore polycarbonate filters using a manual thermostat-heated extrusion device (Avanti^®^ Polar Lipids, Alabaster, AL, USA). The suspension was filtered 20 times through filters with a pore size of 100 nm, at 74°C.

The size and the polydispersity of the vesicles were determined by granulometry by dynamic laser light-scattering measurements using a Malvern High Performance Particle Sizer (HPPS-ET, Instrument SA, UK). Measurements were performed in triplicate after dilution of the suspension in filtered buffer.

Before liposome labeling, untrapped arginine was removed by FPLC (fast protein liquid chromatography) using a size-exclusion Superdex^®^ 200 column (Amersham pharmacia biotech, Orsay, France) eluted in 150 mmol/l, pH 5.6 phosphate buffer.

#### Radiolabeling Procedure

The aqueous phase radiolabeling was obtained by encapsulation of ^125^I using an active-loading method in which radioiodinated BH reagent reacts with pre-encapsulated arginine after crossing the lipid bilayer (12). The resulting positively charged conjugate (^125^I–BH–arginine) is then trapped inside the liposomes. In order to check the integrity of liposomes *in vivo*, they were also radiolabeled on the surface with ^111^In by complexation with DTPA coupled to phospholipids (14). Moreover, the DTPA–In complex constitutes also the hapten specifically recognized by the antibody 734.

Bolton–Hunter reagent was first radiolabeled with ^125^I by the chloramine T method and purified by solvent extraction (12). The organic solvent was then evaporated using a dry nitrogen stream.

Then, the double radiolabeling was obtained in one-step on preformed liposomes. Arginine-containing liposomes were added to the dry ^125^I–BH reagent (90 nmol of reagent for 1 μmol total lipids). Then, citrate buffer (100 mM, pH 5.0) was added, in order to obtain a final citrate concentration of 10 mM and a pH range of 5–6 required for ^111^In membrane radiolabeling. A mixture of a known amount of ^115^InCl_3_ with a trace activity of ^111^InCl_3_ (in HCl 0.06N) was added. Membrane radiolabeling was performed with a ratio of one indium (^115^In + ^111^In) molar equivalent per mole of available DTPA. The activity of ^111^In was used to determine the radiolabeling efficiency and the molar amount of ^115^In bound to DTPA that reflects the number of haptens expressed at the liposome surface. The liposomes were then incubated for 30 min at 37°C with ^125^I–BH and ^111^In. At the end of incubation, a solution of EDTA was added to chelate free indium (10 EDTA molar equivalent per mole of indium) before purification by size-exclusion chromatography using a PD10 column (Sephadex G25, Bio-Rad). The labeling efficiencies were determined after purification by counting the different elution fractions for the two isotopes, with a γ-counter (Wallac 1480-Wizard^®^3, Perkin-Elmer, Paris, France).

#### Fluorescent Liposomes

To investigate the integrity of the DSPC/Chol/DSPE–PEG2000/DSPE–PEG2000–DTPA/DPPE–Rhodamine liposomes, the lipid bilayer of the fluorescent liposomes was labeled with Rhodamine and the aqueous phase with Fluorescein (Rho-labeled Fluo–Arg-loaded liposomes). Fluorescein was used in the form of isothiocyanate (FITC), coupled beforehand with arginine to form fluorescein–arginine (Fluo–Arg) conjugate, and encapsulated in a passive way during the preparation of liposomes, to a concentration of approximately 20 mM.

To be in the same conditions as with the radioactive liposomes and in order to form the DTPA–In hapten, functionalized liposomes were then saturated with non-radioactive ^115^In. This saturation was performed in acetate buffer 10 mM pH 5, by addition of indium chloride (HCl 0.06N) with 10 molar equivalents of ^115^In per mole of DTPA.

### Fluorescence Microscopy

#### Cell Lines

The cell line was the same for *in vitro* and *in vivo* experiments. These human colorectal adenocarcinoma cells (LS174T) were acquired from American Type Culture Collection (USA) and expressed strongly the CEA antigen on their surface. They were cultured in medium suggested by RPMI 1640 (Gibco^®^) supplemented with glutamine 2 mM (Invitrogen, France) and 10% (*v*/*v*) fetal bovine serum (FBS) (Laboratory PAA, France). Cells were grown in tissue culture flasks to confluence at 37°C in humidified atmosphere with a partial pressure of CO_2_ of 5%.

Fluorescence microscopy studies were performed in time-lapse to monitor the kinetics of interaction between cells and liposomes. LS174T cells (100,000/well) were washed once before to replace the culture medium by 300 μl of a diluted solution of BsmAb (10 μg/ml for specific targeting or 0 μg as control). After 1 h of incubation, cells were washed three times by 300 μl of culture medium. Rho-labeled Fluo–Arg-loaded functionalized liposomes were diluted (25 nmol/ml of lipids in RPMI) and 300 μl were incubated with cells, which represents approximately 750,000 liposomes per cell. Acquisitions were performed in time-lapse after 15 min of incubation between liposome suspension and cells, during 6 h. To compare active (specific targeting with BsmAb) and passive targeting (without BsmAb), the fraction of liposomes bound to cells was quantified by measuring the fluorescence intensity of rhodamine.

#### Detection by Fluorescence Microscopy

Imaging of interactions between functionalized liposomes and cells was performed using a Nikon A1 Rsi confocal microscope (objectives Plan Apo ×60/1.4 and Plan Apo ×20/0.75). The microscope is adapted to the confocal imaging of fixed or living cells (control of the temperature or of the rate of CO_2_).

Fluorescence signals of fluoresceine and rhodamine were recorded after excitation by an argon laser and by a diode laser (respectively 488 and 561 nm exciter bandpass filters). The emitted fluorescence was respectively collected at 525 and 595 nm (emitter bandpass filters). The images were acquired in a matrix size of 512 × 512 pixels and analyzed by the Fiji software and the NIS element (Nikon) software.

### Biodistribution Studies

Animal experiments were carried out in compliance with French regulation and approved by the Ethics Committee for animal experimentation – Région Pays de la Loire France (approval number: B44.565) according to the protocol CEEA.2012.171. NMRI-nu (*nu/nu*) mice were purchased from Janvier^®^ (Le Genest Saint Isle, France). Mice were housed under standard conditions (standard diet and water *ad libitum*) and maintained in post-entry quarantine for 2 weeks before experiments.

Tumor targeting was performed in mice with CEA-expressing subcutaneous tumor xenografts. Isolated human colorectal adenocarcinoma LS174T cells (2.5 × 10^6^) in 100 μl of sterile physiologic serum were injected into the right flank of NMRI-nu (*nu/nu*) mice. Biodistribution studies were performed at 10–15 days post-graft according to tumor growth (4–8 mm diameter tumors). Liposomes and antibodies were injected by intravenous bolus injection via the tail vein.

For the active targeting protocol, unlabeled anti-CEA/anti-DTPA–In BsmAb (130 μg in 100 μl PBS, which corresponds to 1.3 nmol) was injected first. Then, selected liposome formulations were injected 24 h after the BsmAb. Mice received 100 μl of doubly radiolabeled liposomes containing 0.037–0.185 MBq (1–5 μCi) with 500 nmol of total lipids/mouse (100 nmol of functionalized liposomes that corresponds to 0.5 nmol of hapten and 400 nmol of non-functionalized liposomes co-injected to saturate the reticulo-endothelial system) (15). Control experiments were performed to determine the passive targeting of liposomes, without injection of the BsmAb, under the same conditions.

Groups of four to five mice were used for each time point of biodistribution study: 3, 24, and 48 h post-injection. At each time point, blood samples were collected just before sacrifice and then tumor and normal organs were dissected. All samples were weighed and counted in a gamma counter calibrated for the two isotopes. Standards of the injected material were made in duplicate and used to calculate the total injected dose and to correct for decay of the radioisotopes. The total radioactivity in the blood was determined by assuming that the total volume of blood was 7% of the mice body weight (16). The results are expressed as a percentage of the total administered liposome dose accumulated per gram of tissues or remained in the blood (% DI/gram) ± SD.

The data from the biodistribution studies were compared using the non-parametric Wilcoxon’s test, due to the small numbers of animals, using *p* = 0.05.

## Results

### Liposomes Preparation and Characterization

After filtration of the liposomes with the extruder, the mean size obtained for the different formulations was 105.5 ± 6.4 nm (polydispersity index <0.1).

The double radiolabeling of the liposomes, by ^125^I–BH encapsulation in the aqueous phase and by ^111^In chelation on DSPE–PEG–DTPA at the surface, was performed by 30 min incubation at 37°C. Around 60% of encapsulation was obtained and the surface radiolabeling efficiency was above 77%. The number of DTPA–In on the liposome surface was estimated using a known amount of ^115^In and a tracer proportion of ^111^In to be around 5 nmol/μmol of lipids.

### Fluorescence Microscopy

**Figure 2:**
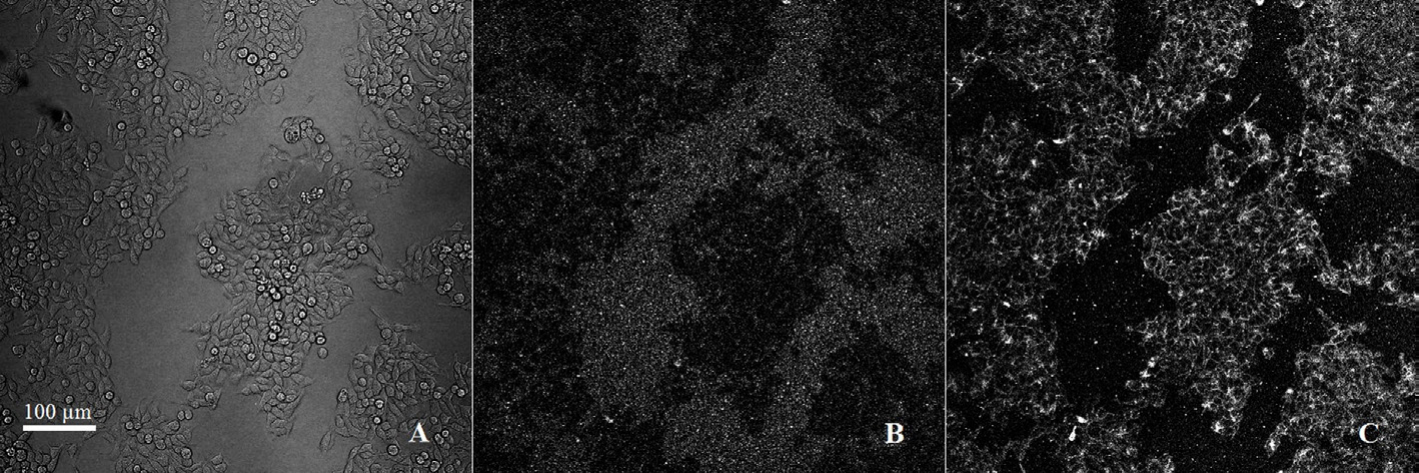
**Observation in time-lapse confocal microscopy (zoom **×**20/640 **μ**m **×** 640 **μ**m): active targeting of doubly fluorescent functionalized liposomes on LS174T cells**. **(A)** cells in brightfield, **(B)** FITC signal (ex/em 488/525 nm), **(C)** rhodamine signal (ex/em 561/595 nm). Scale bar: 100 μm.

The kinetics of binding of the liposomes to the cells were assessed by time-lapse fluorescence microscopy (Figure 2). From the beginning of the acquisition and during 6 h, we observed an intense binding of rhodamine to cell membranes while no fluorescence was observed inside the cells. The membrane-associated fluorescence was very intense and stable over time, but leakage of Fluo–Arg contained in the liposomes occurred when they interacted with the cell surface. Very quickly, a diffuse green fluorescence was observed in the extracellular area.

**Figure 3:**
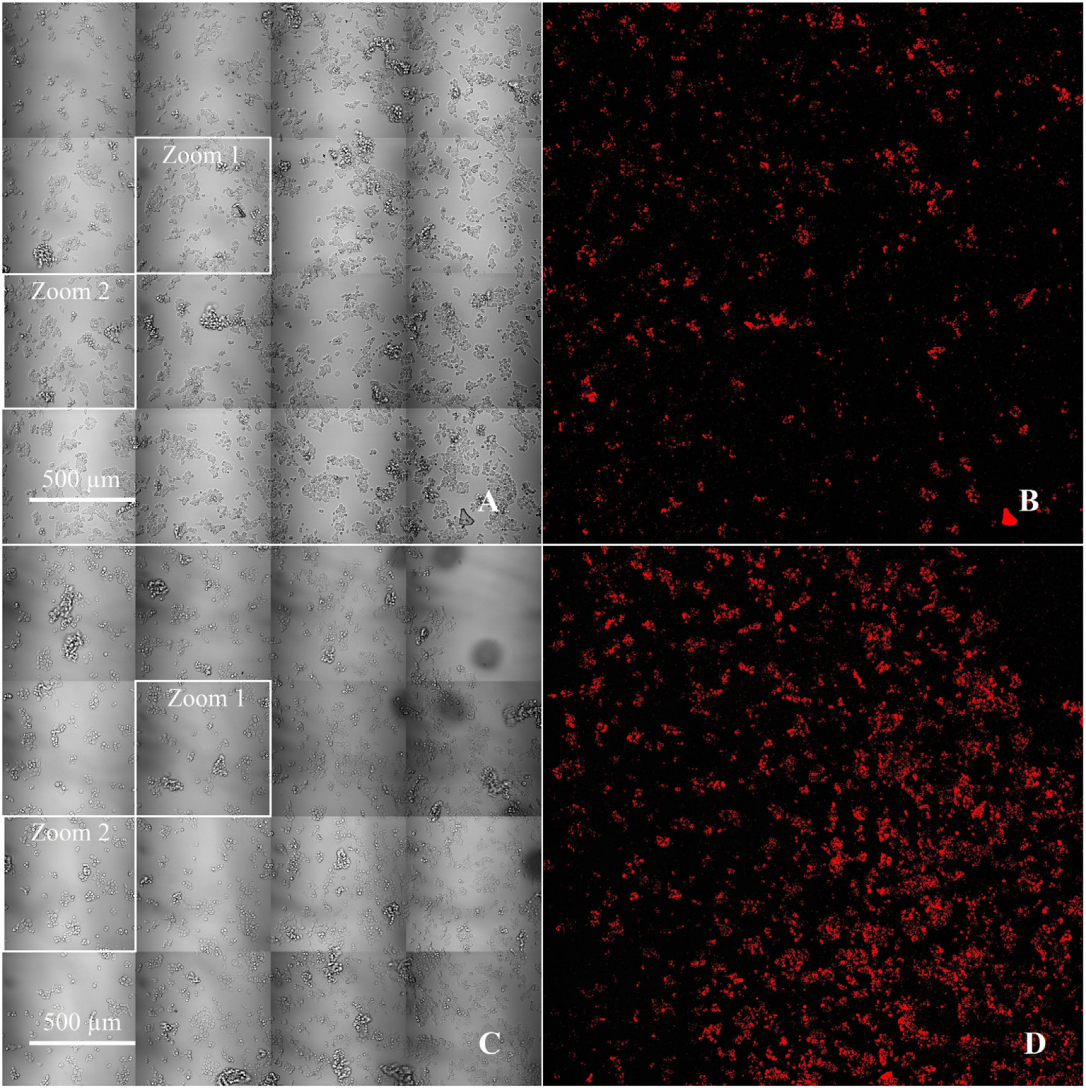
**Comparison between passive (A,B) and active targeting (C,D) of fluorescent functionalized liposomes on LS174T cells, with cells in brightfield (A,C) and the corresponding rhodamine signal thresholded by the Otsu algorithm (B,D) (**×**20/2545 **μ**m **×** 2545 **μ**m)**.

To demonstrate the immunospecificity of functionalized liposome targeting to LS174T cells, acquisitions were performed by comparing the rhodamine signal bound to the membranes, with or without preincubation of cells with the bispecific antibody. In order to remove liposomes unbound to the cells, three washes were carried out by replacing the culture medium after 3 h of incubation with the liposomes. Signal obtained with rhodamine is represented in Figure 3, respectively for active targeting with antibody and passive targeting without antibody. These images represent the amount of fluorescence signal of five consecutive focal planes acquired along the *Z* axis and spaced with 2 μm.

Then, we selected two fields (zoom 1 and zoom 2) in which cells were counted in order to evaluate the fluorescence signal according to the number of cells. The intensity of the signal was thresholded by the Otsu algorithm (Table 1). For zoom 1, the mean fluorescence signal was 103 pixels per cell for active targeting and 29 pixels per cell for passive targeting, a bonding of Rho-labeled liposomes 3.6 times higher for active targeting. For zoom 2, where the difference of fluorescence was the lowest visually between passive and active targeting, the mean signal was 66 and 31 pixels per cell, respectively, for the active and passive targeting, with a ratio of 2.1 for active targeting.

**Table 1:** **Data obtained *in vitro* with rhodamine (lipid bilayer tracer) after quantification of the fluorescent signal on the LS174T cells membrane compared to the number of the cells for active targeting or passive targeting of fluorescent and functionalized liposomes**.

Targeting	16 fields	Zoom 1			Zoom 2		
	Fluorescence (pixels)	Cells	Fluorescence (pixels)	Pixels per cells	Cells	Fluorescence (pixels)	Pixels per cells
Passive targeting	107,498	622	17,929	29	583	17,929	31
Active targeting	331,454	424	43,881	103	343	22,646	66
Ratio	3:1	–	–	3:6	–	–	2:1

*Zoom 1 and Zoom 2 correspond to the selected areas on Figure 3*.

### Biodistribution Experiments

The best formulation of functionalized liposomes was defined in preliminary studies in terms of affinity to the BsmAb, tested by SPR, and in terms of pharmacokinetic parameters *in vivo* (11, 14). The chosen formulation (DSPC/Chol/DSPE–PEG2000/DSPE–PEG2000–DTPA) showed the best affinity with 6.3 nM and favorable pharmacokinetic parameters (T1/2β ≅ 12.5 h). This formulation with DTPA at the end of the PEG chains orientates the hapten away from the PEG structure, in order to avoid steric hindrance.

*In vivo* biodistribution in tumor-xenografted nude mice is represented in Table 2. The activities in blood and in major organs are presented at 3, 24, and 48 h. The blood activities are quite comparable for the two radioisotopes that confirm the integrity of the circulating liposomes *in vivo*. Uptake in major organs was higher at 24 h with a higher accumulation in liver, spleen, and kidneys, as widely described in the literature. Moreover, indium-labeled phospholipids remained in liver and spleen, which reflects the accumulation of radiolabeled phospholipids in catabolizing organs, whereas the radioiodinated BH-arginine is quickly eliminated from blood after liposome destruction. For example, the uptakes in liver, spleen and kidneys are respectively 16.5 ± 2.4, 34.3 ± 3.5, 8.8 ± 1.7% ID/g with ^111^In and 4.1 ± 1.1, 9.1 ± 3.7, 2.1 ± 0.8% ID/g with^125^I.

**Table 2:** **Biodistribution of functionalized DSPC/Chol/DSPE–PEG_2000_/DSPE–PEG_2000_–DTPA–In (64.5:30.5:3.5:1.5) liposomes injected in LS174T-xenografted nude mice (0.5 nmol of hapten/mouse and 1–5 **μ**Ci for each radionuclide: ^111^In and ^125^I) 24 h after bispecific antibody (130 **μ**g hMN14 **×** 734)**.

% ID/g	^111^In			^125^I		
Tissue	3 h (***n*** **=** 4)	24 h (***n*** **=** 4)	48 h (***n*** **=** 4)	3 h (***n*** **=** 4)	24 h (***n*** = 4)	48 h (***n*** = 4)
Tumor	3.6 ± 0.2	7.5 ± 2.4	5.6 ± 1.1	2.5 ± 1.0	5.1 ± 2.5	1.2 ± 0.3
Blood	20.3 ± 2.5	2.9 ± 1.0	0.5 ± 0.2	18.0 ± 3.5	2.1 ± 0.6	0.3 ± 0.1
Liver	11.4 ± 1.1	16.5 ± 2.4	15.3 ± 2.0	6.7 ± 1.3	4.1 ± 1.0	0.5 ± 0.1
Kidneys	6.9 ± 1.0	8.8 ± 1.7	7.6 ± 2.4	3.5 ± 0.7	2.1 ± 0.8	1.1 ± 0.3
Intestine	2.8 ± 1.0	2.7 ± 1.5	3.0 ± 0.6	2.0 ± 0.6	1.4 ± 0.7	0.5 ± 0.2
Lung	7.0 ± 1.1	2.0 ± 0.7	1.3 ± 0.3	5.1 ± 1.4	1.4 ± 0.6	0.25 ± 0.08
Muscle	0.6 ± 0.1	0.5 ± 0.1	0.42 ± 0.09	0.35 ± 0.07	0.16 ± 0.05	0.04 ± 0.01
Spleen	21.2 ± 1.9	34.3 ± 3.5	29.4 ± 2.4	8.7 ± 0.3	9.1 ± 3.7	4.3 ± 2.2
Skin	1.8 ± 0.4	4.2 ± 1.6	4.1 ± 0.5	1.0 ± 0.3	1.6 ± 0.9	0.6 ± 0.1
Brain	1.1 ± 0.4	0.16 ± 0.04	0.08 ± 0.01	0.8 ± 0.3	0.12 ± 0.04	0.014 ± 0.003
Heart	4.8 ± 2.2	2.2 ± 0.7	2.0 ± 0.4	4.8 ± 1.8	1.3 ± 0.4	0.6 ± 0.2
Bone	3.4 ± 0.7	2.0 ± 0.6	1.6 ± 0.2	2.3 ± 0.5	0.6 ± 0.3	0.13 ± 0.04
Stomach	0.8 ± 0.1	0.8 ± 0.5	0.6 ± 0.4	0.66 ± 0.09	0.5 ± 0.3	0.18 ± 0.09

*The data are expressed as the percentage of total administered dose per gram of tissue (% ID/g, mean ± SD)*.

**Figure 4:**
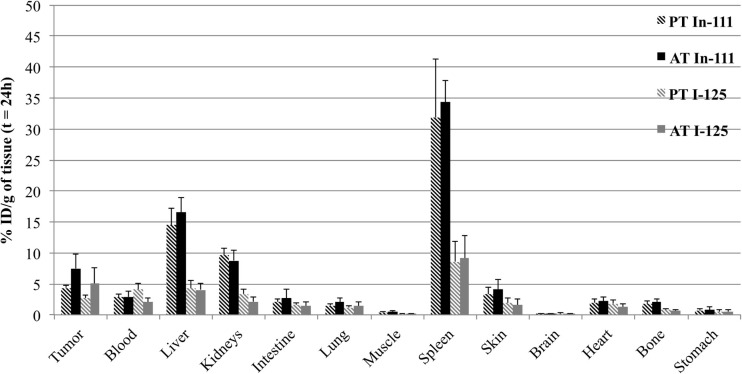
**Biodistribution of doubly radiolabeled liposomes (^111^In in black and ^125^I in gray) at 24 h in LS174T-xenografted nude mice for passive targeting or active targeting**. Mice were injected i.v. with functionalized liposomes (0.5 nmol of hapten/mouse and 1–5 μCi for each radionuclide) without BsmAb injection (PT for Passive Targeting) or 24 h after BsmAb injection (AT for Active Targeting). The data are expressed as the percentage of total administered dose per gram of tissue (% ID/g, mean SD).

Comparison of organ uptake for passive and active targeting is presented in Figure 4, for the two tracers. Results are presented at 24 h, when tumor uptake is highest. Biodistribution in healthy tissues was comparable for active and passive targeting, and no significant difference was shown for liver, spleen, kidneys and blood between passive and active targeting (*p* > 0.05).

Concerning tumor uptake, results showed a relatively important passive targeting of the liposomes, in particular for the tracer of the lipid bilayer, with 4.4 ± 0.4% ID/g of tumor with ^111^In, and 2.7 ± 0.6% ID/g with ^125^I. In spite of this high passive targeting, results demonstrated significant specific targeting, after BsmAb injection, with 7.5 ± 2.4% ID/g of tumor with ^111^In, and 5.1 ± 2.5% ID/g with ^125^I (*p* = 0.03), demonstrating the immunospecificity of the two-step targeting using functionalized liposomes and BsmAb.

On the other hand, it seemed interesting to take into account all the results of the biodistribution, by calculating the areas under the curve (AUC), which integrated the activities at 3, 24, and 48 h (Table 3). The values of AUC were calculated by the trapeze method from the mean activities for each time point. If we compare the results obtained with ^111^In (surface labeling) and ^125^I (internal labeling), we can notice that for the healthy organs, the AUC is two to four times higher for the membrane labeling than for the internal phase labeling. Tumor/organs ratios are 0.4, 0.2, and 0.7 in ^111^In and 0.9, 0.4, and 1.6 in ^125^I, respectively, for liver, spleen, and kidneys. These ratios are more favorable for the internal labeling with values from 1.5 to 2 times superior to the ratios obtained for the membrane labeling.

**Table 3:** **Areas under the curve (AUC) and tumor/organs ratios (T/O) calculated for the main organs (% ID/g) after biodistribution (3, 24, and 48 h) of functionalized liposomes administered i.v. 24 h after BsmAb injection in LS174T-xenografted nude mice**.

	^111^In		^125^I		
	AUC	T/O	AUC	T/O
Tumor	278	1.0	158	1.0
Blood	437	0.6	329	0.5
Liver	692	0.4	178	0.9
Kidneys	371	0.7	103	1.6
Spleen	1379	0.2	361	0.4

## Discussion

The originality of this study was to design a two-step targeting system with liposomes, similar to that used in AES, for the RIT of solid tumors. Once the radiolabeling technique was finalized and the best formulation was selected by SPR (11), we were able to test these liposomes on a cellular model, using CEA-expressing LS174T cells, in order to characterize the specific interactions between liposomes and target cells.

*In vitro*, liposomes were surface-labeled with rhodamine and contained entrapped Fluo–Arg. Indeed, it seemed important in this work to monitor liposome integrity using a tracer of the membrane and a tracer encapsulated in the aqueous phase (17). To observe the kinetics of interaction of the liposomes with the cells, time-lapse fluorescence microscopy was used. The incubation of LS174T cells with Rho-labeled Fluo–Arg-loaded functionalized liposomes did not result in a co-localization of the fluorescent tracers. An intense fixation of the rhodamine on cell membranes was observed during 6 h, while the Fluo–Arg was totally released (diffuse green fluorescence in the extracellular medium). This leakage was attributed to the interaction of the cell surface proteins with the liposome bilayer, and depends on the liposome formulation, in particular when the Chol lipid proportion increased from 30 to 40 moles% of lipids (18).

In this work, despite a supplementation of the medium by 10% of FBS, non-specific binding was observed after incubating the cells and liposomes, without BsmAb (passive targeting). Nevertheless, the rhodamine fluorescence signal associated to the liposomes was higher in the case of active targeting compared to passive targeting with a ratio of 3:1 obtained by signal quantification.

In second part, the ability of functionalized and pegylated liposomes to target CEA-expressing tumors, was tested in xenografted nude mice, with the two-step targeting system using the hMN14 × 734 BsmAb. The double radiolabeling proved of real interest to compare the behavior of the radioactivity encapsulated in the aqueous phase and that carried by radiolabeled phospholipids in the lipid bilayer.

Blood levels of the two tracers remained the same at different times after injection, thus demonstrating the stability of the liposomes in the circulation. By contrast, phospholipids radiolabeled with residualizing ^111^In accumulate in catabolizing organs, with a prominent uptake in liver and spleen, whereas encapsulated-^125^I–BH–arginine was quickly eliminated in urine after liposome destruction (14). The maximum tumor uptake was observed at 24 h. At this time, tumor/organ ratios were superior to 1 for ^125^I, except for the spleen. For liver, spleen and kidneys, ratios were, respectively, 1.24, 0.56, and 2.42 for ^125^I and 0.45, 0.22, and 0.85 for ^111^In.

In spite of relatively high tumor uptake with passive targeting, results demonstrated significant specific targeting, after BsmAb injection, with 7.5 ± 2.4% ID/g of tumor with ^111^In, and 5.1 ± 2.5% ID/g with ^125^I. The lower tumor uptake obtained with ^125^I confirms *in vitro* results. A release of the content was effectively observed by fluorescence microscopy, as the liposomes interacted with the cellular target. Pegylation of the liposomes provides a long half-life, which allows them to reach the tumor, but after interaction with the target cells, the encapsulated-tracer (^125^I–BH–arginine or Fluo–Arg) is released in part. However, encapsulating the radioactivity in the aqueous phase favors a more rapid elimination of the hydrophilic radiolabeled compound and reduces the healthy organs irradiation.

If specific targeting of functionalized liposomes has been documented extensively *in vitro* (17, 19, 20), most of the *in vivo* targeting experiments described in the literature do not show significant differences between passive and active targeting. For example, Petersen et al. described a tumor targeting with a somatostatine analog (TATE)-functionalized liposomes. Results obtained for specific targeting were 5.2 ± 0.5% ID/g versus 5.5 ± 0.3% ID/g with non-specific control liposomes (21). Similarly, no difference in tumor accumulation was obtained using folate-functionalized liposomes to target different tumors which overexpressed folate receptors (22). In this particular case, it was shown that despite the enhanced affinity of specific liposomes to tumor cells, tumor uptake was not improved because of a rapid elimination of the functionalized liposomes by the liver. By using immunoliposomes, carrying monoclonal antibodies or their fragments, specific targeting does not improve tumor accumulation, essentially resulting from the well known enhanced permeability and retention (EPR) effect (23), but nevertheless authors conclude that immunoliposomes can increase interaction with the cells, and notably by internalization (24). On the other hand, no significant difference was demonstrated between passive targeting and active targeting, when the target antigen is internalizing, either with immunoliposomes or with pretargeting (25). The pretargeting system used in our study is based on the recognition of CEA antigen, widely known to be very slowly internalized and not to promote the endocytosis of liposomes (26, 27). In this system, a specific tumor targeting, *in vitro* and *in vivo*, was thus demonstrated with the pretargeted functionalized liposomes.

## Conclusion

Specific targeting of liposomes is difficult to demonstrate *in vivo* because they accumulate in tumors passively through the EPR effect. Nevertheless, this study shows the interest of a two-step targeting, using functionalized liposomes and BsmAb, of a non-internalizing antigen. Indeed, the specific targeting observed *in vitro*, on CEA-expressing cells, is confirmed *in vivo* by a significant increase of tumor uptake *in vivo*, which was not previously described in the literature. The immunospecificity of targeting is indeed often offset by the EPR effect and by the rapid elimination of the immunoliposomes or by the internalization of the antibody used in the pretargeting system. In this work, the pretargeting approach allowed us to use stable unlabeled BsmAb and liposomes tailored for optimal *in vivo* behavior and suitable for extemporaneous radiolabeling. This original and new approach suggests a potential for immunospecific liposome pretargeting for RIT.

## Conflict of Interest Statement

The authors declare that the research was conducted in the absence of any commercial or financial relationships that could be construed as a potential conflict of interest.
